# Halofuginone inhibits colorectal cancer growth through suppression of Akt/mTORC1 signaling and glucose metabolism

**DOI:** 10.18632/oncotarget.4376

**Published:** 2015-06-08

**Authors:** Guo-Qing Chen, Cheng-Fang Tang, Xiao-Ke Shi, Cheng-Yuan Lin, Sarwat Fatima, Xiao-Hua Pan, Da-Jian Yang, Ge Zhang, Ai-Ping Lu, Shu-Hai Lin, Zhao-Xiang Bian

**Affiliations:** ^1^ Laboratory of Brain and Gut Research, Center for Clinical Research on Chinese Medicine, School of Chinese Medicine, Hong Kong Baptist University, Hong Kong SAR, China; ^2^ Chongqing Academy of Chinese Materia Medica, Chongqing, China; ^3^ Department of Chemistry and State Key Laboratory of Environmental and Biological Analysis, Hong Kong Baptist University, Hong Kong SAR, China; ^4^ Instrument and Testing Center, Sun Yat-Sen University, Guangzhou, China; ^5^ Shen Zhen People's Hospital, Shenzhen, China

**Keywords:** halofuginone, anticancer activity, colorectal cancer, Akt/mTORC1, glucose metabolism

## Abstract

The Akt/mTORC1 pathway plays a central role in the activation of Warburg effect in cancer. Here, we present for the first time that halofuginone (HF) treatment inhibits colorectal cancer (CRC) growth both *in vitro* and *in vivo* through regulation of Akt/mTORC1 signaling pathway. Halofuginone treatment of human CRC cells inhibited cell proliferation, induced the generation of reactive oxygen species and apoptosis. As expected, reduced level of NADPH was also observed, at least in part due to inactivation of glucose-6-phosphate dehydrogenase in pentose phosphate pathway upon HF treatment. Given these findings, we further investigated metabolic regulation of HF through Akt/mTORC1-mediated aerobic glycolysis and found that HF downregulated Akt/mTORC1 signaling pathway. Moreover, metabolomics delineated the slower rates in both glycolytic flux and glucose-derived tricarboxylic acid cycle flux. Meanwhile, both glucose transporter GLUT1 and hexokinase-2 in glycolysis were suppressed in CRC cells upon HF treatment, to support our notion that HF regulates Akt/mTORC1 signaling pathway to dampen glucose uptake and glycolysis in CRC cells. Furthermore, HF retarded tumor growth in nude mice inoculated with HCT116 cells, showing the anticancer activity of HF through metabolic regulation of Akt/mTORC1 in CRC.

## INTRODUCTION

More than 1.2 million cases are diagnosed with colorectal cancer (CRC) every year, and more than 600 000 die from the disease worldwide. CRC is responsible for 8% of all cancer deaths [1]. In United States, CRC is the second leading cause of death from cancer among adults [2]. As evidenced by genetic modifications, environmental impacts, diet and lifestyles, the underlying mechanisms of CRC have been intensively studied. Akt, also known as protein kinase B (PKB), which is overexpressing in a number of cancers including colorectal cancer, plays a key role in multiple cellular processes such as glucose metabolism, apoptosis, cell proliferation, transcription and cell migration [3]. Akt activates an array of downstream factors through phosphorylation and then regulates cellular metabolism which is rewired in cancer cells [4]. Of note, mammalian target of rapamycin complex 1 (mTORC1), is led by Akt through phosphorylation at Ser 2448. It has been reported the PI3K/Akt/mTOR signaling components were highly activated in glandular elements of colorectal carcinoma and colorectal adenomas with high-grade intraepithelial neoplasia [5], indicating that inhibitors of PI3K/Akt/mTOR signaling may serve as potential anti-CRC agents.

The cellular metabolism could be regulated by Akt/mTORC1 and the downstream effectors. As a central activator of Warburg effect, mTORC1 can induce glycolytic enzymes in cancer cells [6]. The p70S6K controls lipid/sterol synthesis and switches glucose metabolism from glycolysis to pentose phosphate pathway (PPP) in cancer cells [7]. It is well known that oxidative arm of PPP is one of the major pathways for NADPH (the reduced form of nicotinamide adenine dinucleotide phosphate) production. NADPH is a strong reducing agent which can react with reactive oxygen species (ROS), allowing cancer cells to escape apoptosis [8]. Thus, activated p70S6K regulates redox state of cancer cells and promotes cancer cell growth. 4EBP1 is another well characterized mTORC1 downstream factor which inhibits the initiation of protein translation by binding and inactivating eIF4E (eukaryotic translation initiation factor 4E). Active mTOR regulates 4EBP1 inhibition, increasing glucose uptake and glycolysis [9]. Therefore, PI3K/Akt/mTOR signaling pathway is thought to be very important for glucose and lipid metabolism in cancer cells. This pathway activates aerobic glycolysis (Warburg effect), leading to glucose being avidly consumed by cancer cells for energy demands and survival [10, 11].

**Figure 1:**
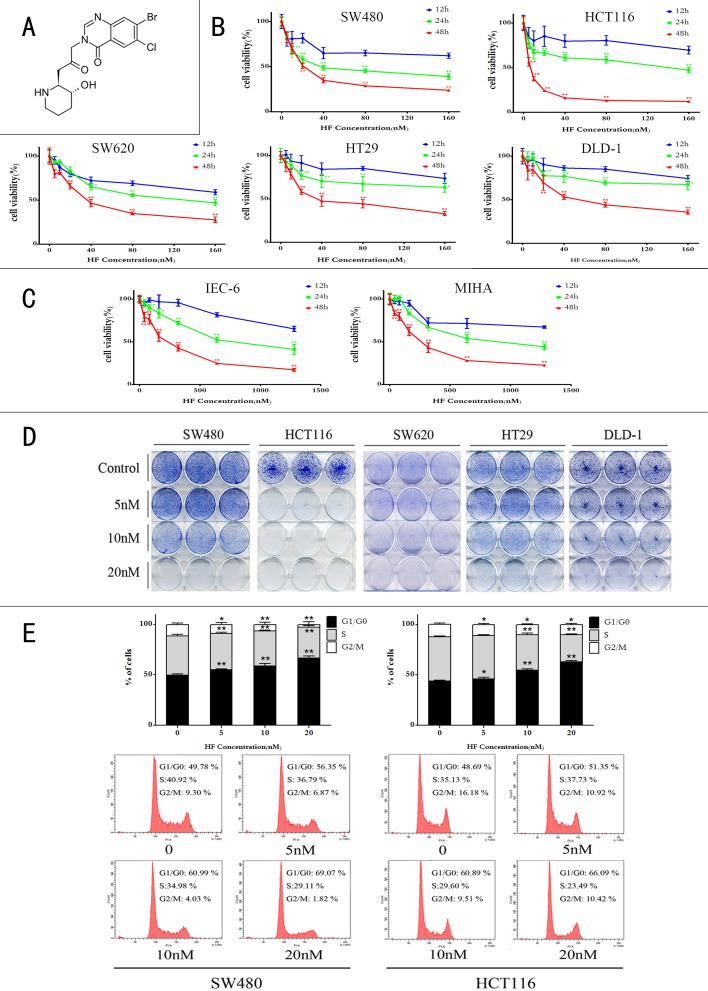
Halofuginone inhibits colorectal cancer cell proliferation **A.** Chemical structure of halofuginone. **B.** MTT assay of five CRC cell lines (SW480, HCT116, SW620, HT29 and DLD-1) treated with increasing concentrations of HF in a time course (12 h, 24 h and 48 h). *P < 0.05, **P < 0.01, compared with 12 h at the same concentration. **C.** MTT assay of non-transformed rat small intestinal epithelial cell line IEC-6 and human non-tumorigenic liver cell line MIHA treated with increasing concentrations of HF in a time course (12 h, 24 h and 48 h). **D.** Colony formation assay of CRC cell lines (SW480, HCT116, SW620, HT29 and DLD-1) treated with 0, 5, 10 and 20 nM of HF. **E.** A flow cytometry analysis of SW480 and HCT116 treated with 0, 5, 10 and 20 nM of HF for 12 h inducing cell cycle arrest in G1/G0 phase. *P < 0.05, **P < 0.01, compared with control group.

Halofuginone (HF) is a derivative of the febrifugine which can be extracted from the Chinese herb *Dichroa febrifuga* Lour. (Changshan in Chinese) (Figure 1A) [12]. It has been used as anti-coccidial drug in animal husbandry for many years [13]. In the last two decades, HF has gained attention for its potential therapeutic effects in fibrotic disease by inhibiting alpha-1 type I collagen gene expression and collagen synthesis [14]. HF also has anticancer activity in bladder carcinoma by inhibiting tumor growth and metastasis through matrix metalloproteinase-2 [15, 16], prostate cancer [17], hepatocellular carcinoma [18], and also modulates the transforming growth factor beta (TGF-β) signaling pathway to inhibit acute promyelocytic leukemia as well as melanoma bone metastases [13, 19]. Particularly, HF has also been found to inhibit Th17 cell differentiation by activating the amino acid starvation response for regulating Stat3-dependent Th17 effector function and reducing established autoimmune inflammation [20, 21]. However, to our best knowledge, whether HF inhibits tumor growth in CRC has not been reported. The aim of this study, therefore, is to investigate the anticancer activity of HF in CRC through the inhibition of Akt/mTORC1 signaling both *in vitro* and *in vivo*. Glucose metabolism and lipid profiles mediated by Akt/mTORC1 signaling pathway in CRC cells upon HF treatment were also depicted by applying metabolomics and lipidomics based on mass spectrometry.

## RESULTS

### Halofuginone treatment inhibits proliferation and induces cell cycle arrest in G1/G0 phase

The effect of HF in CRC cells was examined by performing MTT, colony formation, cell cycle and apoptosis assays as well as measurement of ROS and NADPH levels. As shown in Figure 1B, treatment with increasing concentrations (5 to 160 nM) of HF reduced CRC cell viability compared with the untreated control cells in time- and dose-dependent manners. A sharp decline in cell viability was also observed with increase of HF concentrations. The IC_50_ values were calculated to be 24.83 nM, 5.82 nM, 40.76 nM, 47.61 nM and 60.89 nM for SW480, HCT116, SW620, HT29 and DLD-1 respectively, after 48 h-treatment. In addition, to further test HF toxicity in normal cells, we used non-transformed rat small intestinal epithelial cell line IEC-6 and human non-tumorigenic liver cell line MIHA treated with HF, and calculated IC_50_ values as 205.7 nM and 261.9 nM for IEC-6 and MIHA respectively, after 48 h-treatment, indicating that HF exerts relatively low toxicity to these two non-tumorigenic cell lines (Figure 1C). Colony formation assay was also conducted for examining the effect of HF on CRC cell survival *in vitro*. HF treatment dose-dependently reduced cancer cell survival rates (Figure 1D). Furthermore, the effect of HF on cell cycle progression was tested in SW480 and HCT116 cell lines by PI staining, followed by FACS analysis. As shown in Figure 1E, after HF treatment for 12 h, a dose-dependent arrest occurred in the G1/G0 phase of the CRC cells, indicating that the inhibitory effect of HF on CRC cell proliferation might be correlated with the arrest of G1/G0 cell cycle progression.

### Halofuginone induces apoptosis, increases ROS and decreases NADPH levels

**Figure 2:**
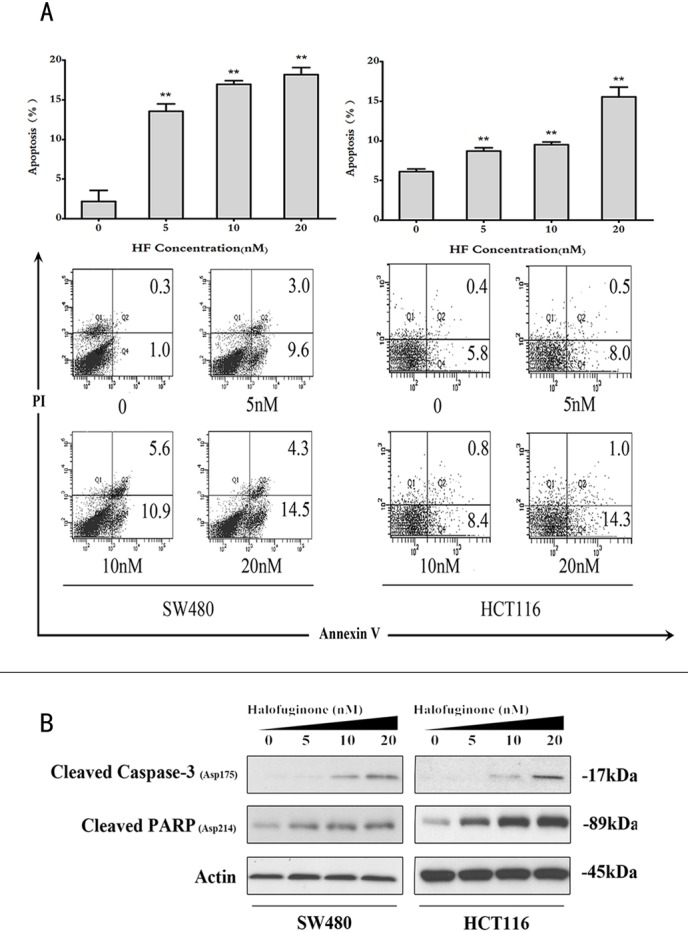
Halofuginone induces cell apoptosis **A.** The bar chart shows the percentage of apoptotic cells in SW480 and HCT116 cell lines treated with 0, 5, 10 and 20nM of HF (upper panel), and the representative flow cytometry annexin V-PI data (lower panel). *P < 0.05, **P < 0.01, compared with control group. **B.** Protein expressions of cleaved Caspase-3 and cleaved PARP in SW480 and HCT116 cell lines treated with 0, 5, 10 and 20 nM of HF.

**Figure 3:**
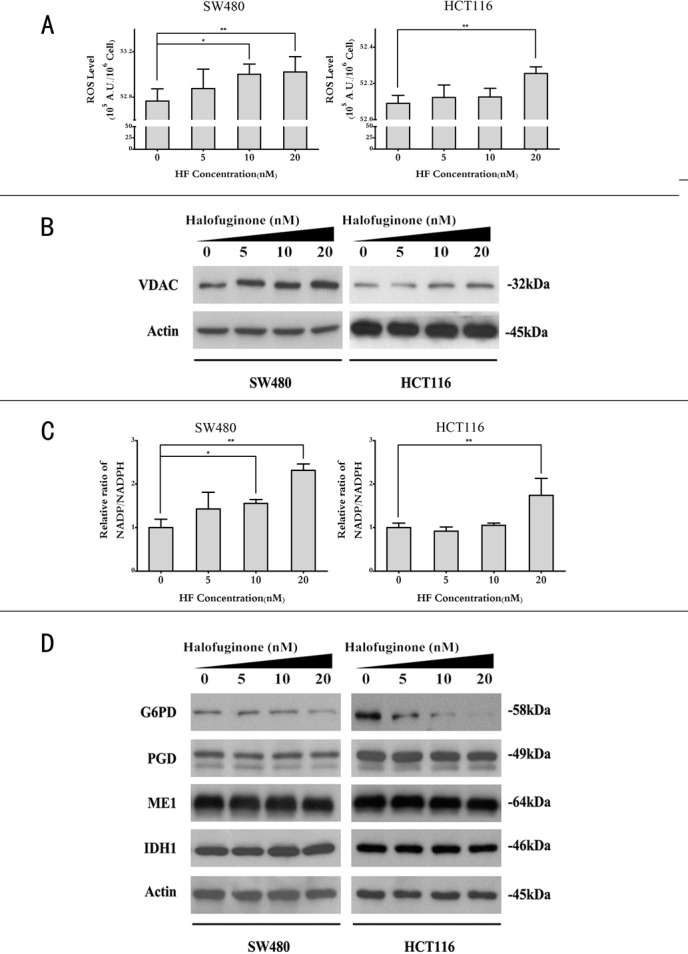
Halofuginone enhances ROS levels while reduces NADPH production **A.** ROS levels in SW480 and HCT116 cell lines treated with 0, 5, 10, 20 nM of HF. *P < 0.05, **P < 0.01, compared with control group. **B.** Protein expression of mitochondrial marker VDAC in SW480 and HCT116 cell lines treated with 0, 5, 10, 20 nM of HF. **C.** Relative ratios of [NADP^+^]/[NADPH] in SW480 and HCT116 cell lines treated with 0, 5, 10, 20 nM of HF. *P < 0.05, **P < 0.01, compared with control group. **D.** Protein expression of G6PD, PGD, ME1 and IDH1 in SW480 and HCT116 cell lines treated with 0, 5, 10, 20 nM of HF (G6PD: glucose-6-phosphate dehydrogenase, PGD: 6-phosphogluconate dehydrogenase, ME1: malic enzyme, IDH1: isocitrate dehydrogenase).

In the cell cycle distribution analysis, sub-G1 peaks in two cell lines also revealed that HF could induce apoptosis (Figure S1). To further determine the effect of HF-induced apoptosis, annexin V/PI dye staining was performed for quantitative analysis of the apoptotic cell percentage in both cell lines treated with increasing concentrations of HF for 12 h. As shown in Figure 2A, HF treatment increases the percentage of both early and late apoptotic cells compared with the untreated control. Consistently, HF treatment profoundly increased the expression of cleaved Caspase-3 and cleaved PARP in a dose-dependent manner as demonstrated by Western blot analysis (Figure 2B). The induction of apoptosis, at least in part due to ROS generation, has been implicated [22]. Therefore, we tested whether HF treatment can enhance ROS formation in CRC cells. After HF treatment for 12 h, CRC cells produced more ROS than control group (Figure 3A). In particular, 20 nM HF pronouncedly elevated ROS levels in both SW480 and HCT116 cell lines. Co-treatment with N-Acetyl-L-cysteine (NAC) fully reversed the HF-induced increase in ROS and cell death (Figures S2 and S3). To further evaluate mitochondria-derived ROS production, we detected mitochondrial marker voltage-dependent anion channel (VDAC) located on the outer mitochondrial membrane and found that HF treatment markedly augmented VDAC protein levels (Figure 3B). Based on the ROS results, we also measured the reducing power NADPH production and observed the increased ratios of [NADP^+^]/[NADPH] in both SW480 and HCT116 cell lines in agreement with elevated ROS levels (Figure 3C). To further identify which metabolic enzyme generating NADPH is affected by HF treatment, glucose-6-phosphate dehydrogenase (G6PD, EC: 1.1.1.49) and 6-phosphogluconate dehydrogenase (PGD, EC: 1.1.1.44) in oxidative branch of PPP, malic enzyme (ME1, EC: 1.1.1.40) in pyruvate metabolism and isocitrate dehydrogenase (IDH1, EC: 1.1.1.42) in tricarboxylic acid (TCA) cycle were examined. Surprisingly, only G6PD was pronouncedly downregulated, which could attenuate the power of scavenging ROS (Figure 3D and Figure S4).

### Halofuginone downregulates Akt/mTORC1 signaling pathway, slows glycolysis and inhibits lipid biosynthesis

**Figure 4:**
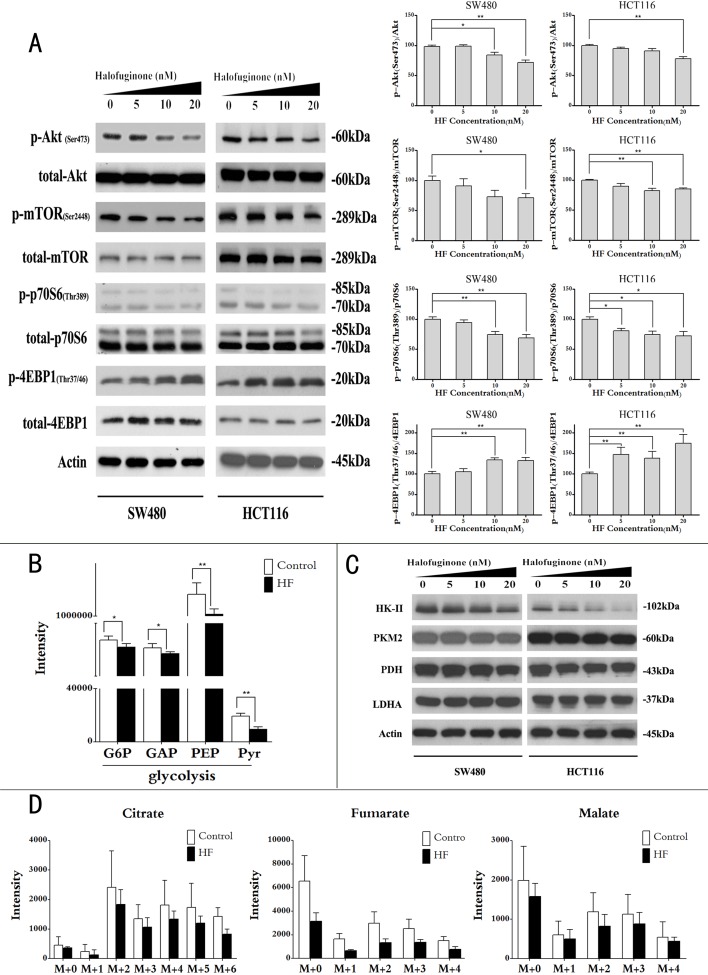
Halofuginone suppresses Akt/mTORC1 signaling pathway and slows glycolytic flux and glucose-derived TCA cycle flux **A.** Protein expressions of phosphorylation of Akt, mTORC1, p70S6 and 4EBP1 (left panel); quantitative analysis of protein expressions (right panel) in SW480 and HCT116 upon HF treatment in a dose-dependent manner for 12 h. *P < 0.05, **P < 0.01, compared with control group. **B.** Uniformly ^13^C-labeled glucose feeding cancer cells for intermediate metabolites in glycolysis by using UPLC-MS/MS (G6P: glucose-6-phosphate, GAP: glyceraldehyde-3-phosphate, PEP: phosphoenolpyruvate, Pyr: pyruvate). The results shown are means ± SEM, *n* = 5. *P < 0.05, **P < 0.01, compared with control group. **C.** Protein expressions of HK-II, PKM2, PDH and LDHA by Western blot (HK-II: hexokinase-2, PKM2: M2 isoform of pyruvate kinase, PDH: pyruvate dehydrogenase, LDHA: lactate dehydrogenase A). **D.** The GC/MS analysis of [U-^13^C_6_]-glucose contribution to citrate, fumarate and malate synthesis in HCT116 cell with or without HF treatment. All GC/MS data were corrected for natural abundance isotopic contribution and normalized to cell number and internal standard 4-chloro-DL-phenylalanine. The results shown are means ± SEM, *n* = 5.

As mentioned above, HF inhibited cell proliferation, induced ROS and apoptosis. We also reasoned how HF treatment regulates metabolic pathways to exert anticancer activity against CRC. Given that Akt/mTORC1 pathway plays a central role in the activation of Warburg effect for cancer cell survival [6, 23], we further asked whether HF could repress aerobic glycolysis via Akt/mTORC1 signaling pathway in CRC cells. After treatment for 12 h, HF resulted in decreased phosphorylation of Akt, mTORC1 and p70S6K while increased phosphorylation of 4EBP1 in a dose-dependent manner (Figure 4A). To further delineate glycolytic flux and glucose-derived TCA cycle flux in CRC cells, we used [U-^13^C_6_]-glucose as an isotopic tracer in feeding cancer cells with or without HF treatment for 12 h. Metabolomics in combination with isotope labeling technique by using ultrahigh-performance liquid chromatography tandem mass spectrometry revealed reduced intermediate metabolites in glycolysis (Figure 4B). To further test which glycolytic enzyme is affected by HF treatment, we assayed protein expressions of hexokinase 2 (HK-II), M2 isoform of pyruvate kinase (PKM2), pyruvate dehydrogenase (PDH) and lactate dehydrogenase A (LDHA). Intriguingly, only sharp decrease of HK-II was observed (Figure 4C and Figure S5). The isotopic patterns of intermediate metabolites in TCA cycle was also analyzed by using gas chromatography mass spectrometry. As a result, the reduced intermediate metabolites in TCA cycle were observed upon HF treatment in HCT116 cells. Scrambling of carbon from glucose was detected in citrate, fumarate and malate with different scrambled labeling patterns (e.g., m+2 and m+4 isotopologues in Figure 4D). Interestingly, the m+2 isotopologues of these three metabolites are the most abundant in the isotopologues of each metabolite because acetyl-Coenzyme A labelled with two ^13^C enters TCA cycle from glycolytic shunt. Due to a broad effect correlated with glucose metabolism, we further investigated the possibility of glucose uptake altered by HF treatment. As a result, glucose transporter GLUT1 was found to be downregulated in CRC cells upon HF treatment (Figure S6), indicating that glucose uptake was also inhibited as an upstream event. In addition, it is well known that glucose is one of the main carbon sources for lipid biosynthesis which could be regulated by p70S6K. Thus, we further profiled lipid species of cancer cells under influence of HF treatment by using ultrahigh-performance liquid chromatography Orbitrap XL mass spectrometry. As a result, up to 49 lipid species in HCT116 cells covering phosphatidylcholine, ceramide, sphingomyelin, phosphatidylglycerol, phosphatidylethanolamine, phosphatidylserine, phosphatidylinositol and phosphatidic acid were downregulated by HF treatment (Figure S7). All of the lipids were tentatively identified by highly accurate masses with mass error of less than 3 ppm in high resolution mass spectrometry by searching against METLIN Metabolite Database. The lipid profiling was consistent with lower expression of fatty acid synthase (FAS) which is an enzyme encoded by the FASN gene in human (Figure S8).

### Halofuginone treatment retards tumor growth in xenograft-bearing nude mice

**Figure 5:**
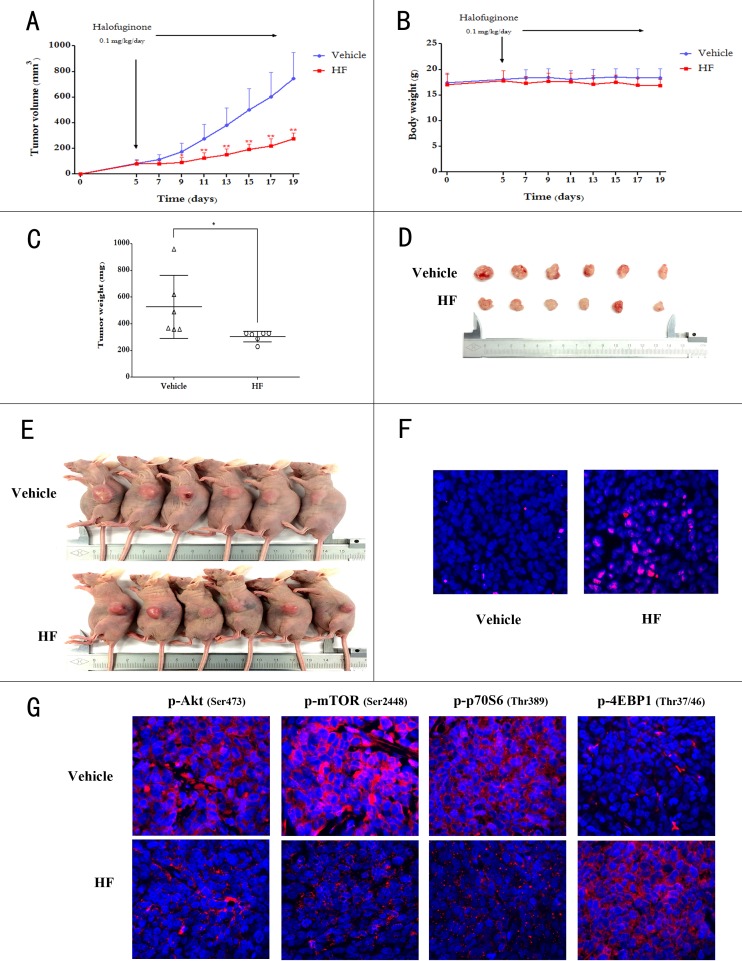
Halofuginone retards tumor growth in xenograft-bearing BALB/c nude mice **A.** Tumor volumes between HF-treated and vehicle group during treatment for 14 days. *P < 0.05, **P < 0.01, compared with vehicle group. **B.** Change in body weight between HF-treated and vehicle group. **C.** Change in tumor weight between HF-treated and vehicle group. *P < 0.05 compared with vehicle group. **D.** The xenograft tumors were dissected and measured after two weeks and shown. **E.** Photos of all of the animals. **F.** TUNEL staining of paraffin embedded 5 micron think tumor sections of HCT116 xenograft-bearing nude mice. **G.** The decreased p-Akt, p-mTORC1, p-p70S6K and increased p-4EBP1 are shown in the immunofluorescence staining.

Next, we tested the ability of HF to retard tumor growth *in vivo.* Nude mice were subcutaneously injected with HCT116 cells and on 5^th^ day after tumor inoculation the mice daily received saline or HF (0.1 mg/kg/day) intraperitoneally for 14 consecutive days. After 14 days of administration, the animals were euthanized to compare tumor burden between vehicle (saline treatment) and HF-treated groups. The HF treatment significantly retarded tumor growth in nude mice as measured by tumor volume while body weight was unaffected (Figures 5A and 5B). Moreover, tumor weight and size in HF-treated group are markedly less than the vehicle group (Figures 5C, 5D and 5E). To further define the mechanism of tumor growth inhibition *in vivo*, the TUNEL apoptotic assay was performed on tumor tissues. As can be seen in Figure 5F, TUNEL staining showed that HF treatment induced significant apoptosis in tumors, as shown by the stronger red fluorescence labeled anti-BrdU monoclonal antibody. We also validated the Akt/mTORC1 signaling pathway *in vivo* upon HF treatment. The visualization indicated decreased staining of phosphorylated Akt, mTORC1 and p70S6K, and increased staining of phosphorylated 4EBP1 in HF treated group compared to the vehicle group (Figure 5G and Figure S9), in line with the *in vitro* results.

## DISCUSSION

Colorectal cancer (CRC) is a malignant neoplasm affecting the lower gastrointestinal tract, awaiting novel drugs for selectively killing CRC [24]. In the functional examination of this study, we found that HF has high toxicity to CRC cells with the lowest concentration of 5.82 nM as IC_50_ in HCT116 cells after 48 h-treatment. But on the other hand, HF displays relatively low toxicity to the tested non-tumorigenic intestinal epithelial cells or liver cells by MTT assay. This compound exerted anticancer activity through inhibition of cancer cell proliferation, induction of ROS and apoptosis *in vitro*, in line with the effect of HF in breast cancer [25]. In CRC cells, HF-induced cell death is rescued by the antioxidant NAC. Additionally, the augmented expression of mitochondrial marker VDAC in SW480 and HCT116 cell lines indicated that the elevated mitochondria-derived ROS production might contribute to cell apoptosis, as previously reported [26-29]. As expected, the reducing power NADPH significantly decreased in SW480 and HCT116 cell lines upon HF treatment, which might be at least partially caused by the inhibition of G6PD [30]. G6PD, as the first and rate-limiting enzyme of the PPP, can be inactivated to reduce NADPH production and biosynthesis, like tumor suppressor gene *p53* [31]. Therefore, HF can also dampen oxidative branch of PPP which is stimulated by p70S6K [7]. In this regard, how HF guards glucose metabolism should be further interrogated for a better understanding of the underlying metabolic mechanism.

**Figure 6:**
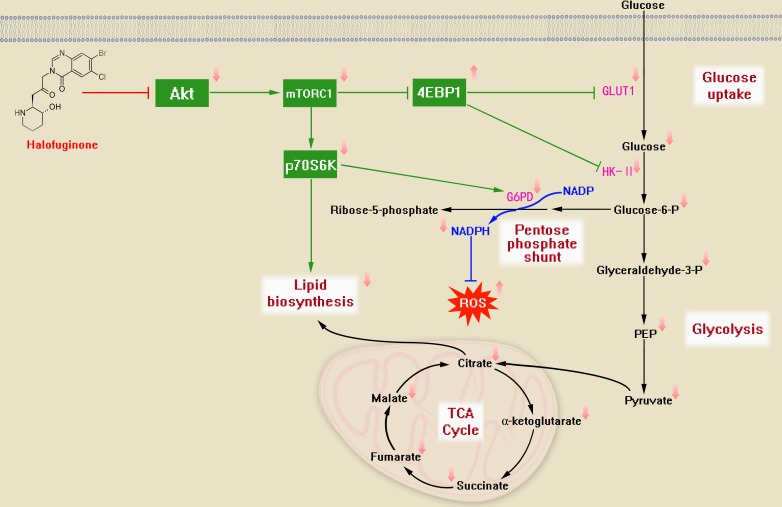
The proposed metabolic mechanism modulated by HF treatment in CRC cells HF can downregulate Akt/mTORC1 signaling pathway and repress Warburg effect, particularly, inhibit GLUT1 and HK-II through activation of 4EBP1. Accordingly, the glycolytic flux and TCA cycle flux are downregulated. Meanwhile, HF can inhibit the downstream of mTORC1 p70S6K for dampening PPP and lipid biosynthesis.

Akt/mTORC1 pathway is a crucial player in CRC development and some of the inhibitors of mTORC1 has been tested in clinical trials [32], suggesting that inhibition of Akt/mTORC1 would be an attractive target in CRC therapy. As one of the main signaling pathways, Akt/mTORC1 signaling triggers Warburg effect and mitochondrial dysfunction in cancer cells [33-35]. In other words, oncogenic processes give rise to rewired metabolism focused upon Warburg effect and the pentose shunt, providing higher levels of reducing activities for cancer cell proliferation, like oncogenic effects mediated by Nrf2, TAp73 [36, 37]. In CRC, glucose consumption and lipid metabolism have also been promoted by oncogenic alterations for tumor growth [38, 39]. In this regard, the anticancer activities of ideal drugs are expected to inhibit oncogenic effects as well as suppress glucose metabolism and lipid biosynthesis in cancer cells. Therefore, we further investigated how HF treatment modulates Warburg effect via Akt/mTORC1 signaling pathway. Of particular interest is that HF could downregulate Akt/mTORC1 pathway in both SW480 and HCT116 cell lines. More importantly, the downstream effectors of mTORC1 were also modulated by HF treatment in CRC cell lines. The p70S6K is one of the effectors of mTORC1, which can activate PPP; while another effector 4EBP1 inhibits glucose uptake and glycolysis. Phosphorylation of p70S6K was downregulated while phosphorylation of 4EBP1 was upregulated, coupling with slower glycolytic rate, suggesting that HF exerts anti-CRC through suppression of Warburg effect. The reduced glucose-derived TCA cycle flux suggested that carbon source from glucose has been significantly inhibited by HF treatment. Surprisingly, glucose transporter GLUT1 and the enzyme HK-II which catalyzes the first committed step of glycolysis, were pronouncedly inhibited by HF treatment in a dose-dependent manner, revealing that the upstream events of glucose metabolism could be mediated by Akt/mTORC1 signaling upon HF treatment. Regarding the growing evidence that HK-II mediates Warburg effect [40] and HK-II is phosphorylated by Akt associated with mitochondria [33], HF treatment in CRC could repress Warburg effect and might also disturb mitochondrial metabolism. An intriguing study shows that tumor initiation and maintenance of lung cancer and breast cancer can be inhibited by ablating HK-II using conditional knockout mice [41], postulating that HK-II could be a key target for HF in treating cancers. Therefore, how HF treatment exactly modulates glucose uptake and HK-II in CRC still needs to be further explored. In addition, lipid biosynthesis mediated by p70S6K activity in Akt/mTORC1 pathway was also found to be downregulated upon HF treatment in CRC cells. Indeed, lipid biosynthesis or lipid droplet formation plays a crucial role in cell proliferation and tumor growth [42-44], which tightly links to mTOR signaling [45-47]. In short, HF could downregulate p70S6K activity for suppression of oxidative PPP and lipid biosynthesis. Together, we propose the metabolic mechanism in CRC cells treated with HF for its anticancer activity (Figure 6).

Inhibitory effects of HF on tumor growth were also validated in xenograft-bearing nude mice. The Akt/mTORC1 signaling pathway was confirmed as the metabolic mechanism of this compound in CRC. Notably, in the animal model, there was no significant change in body weight in HF-treated mice compared to vehicle group, indicating that HF exerts low toxicity to normal cells which was also demonstrated by MTT assay in IEC-6 and MIHA cell lines. In recent studies, HF was found to inhibit phosphorylation of Smad3 via PI3K/Akt and MAPK/ERK pathways in muscle cells [48], and HF can block TGF-β signaling for inhibition of the establishment and progression of melanoma bone metastases [19]. These reports indicate that the inhibition of Akt/mTORC1 signaling would not be the sole pathway functioned by HF. Nonetheless, a highlight of our findings with HF was its activity to inhibit cell proliferation and retard tumor growth both *in vitro* and *in vivo* through metabolic transformation involving the Akt/mTORC1 pathway. Thereby, the Akt/mTORC1 signaling pathway altering glucose metabolism and lipid biosynthesis could be targeted by HF treatment in CRC.

In summary, HF was used to treat CRC for the first time in cells and animal models. The Akt/mTORC1 signaling pathway coupling with metabolic pathways was elucidated to indicate that HF inhibits cell proliferation and induces apoptosis, at least partially due to, the inhibition of Akt/mTORC1. Particularly, GLUT1 and HK-II were found to be markedly inhibited in a dose-dependent manner, which might be associated with metabolic reprogramming modulated by HF treatment. Furthermore, the experiments in nude mice inoculated with human CRC cells validated the therapeutic effect of HF, providing direct evidence to support our notion that HF exerts anticancer activity via Akt/mTORC1 signaling pathway. In conclusion, our study reveals potential therapy of HF against CRC.

## MATERIALS AND METHODS

### Chemicals and reagents

Halofuginone hydrobromide, 2′, 7′-dichlorodihydrofluorescin diacetate (DCFH-DA), Collagenase A, Dnase I, HPLC-grade acetonitrile, HPLC-grade methanol, HPLC-grade isopropanol, Methyl tertbutyl ether (MTBE), Ammonium acetate, N-Acetyl-L-cysteine, acetic acid, Dulbecco's Modified Eagle's Medium without glucose, L-glutamine, phenol red, sodium pyruvate and sodium bicarbonate (DMEM, D5030) and Pierce (R) BCA Protein Assay Kit and [NADP^+^]/[NADPH] Quantification Kit were obtained from Sigma-Aldrich (Munich, Germany). Rat EGF was purchased from Pepro Tech (Rocky Hill, USA). [U-^13^C_6_]-glucose was purchased from Cambridge Isotope Laboratories (Tewksbury, USA). FITC Annexin V Apoptosis Detection Kit I was obtained from BD Bioscience (USA). Dialyzed Fetal Bovine Serum (US Origin SH30079.03) was purchased from HyClone (USA). In situ BrdU-red DNA fragmentation (TUNEL) assay kit (ab66110) was obtained from Abcam.

### Cell culture

HCT116, SW480, SW620, HT29, DLD-1, IEC-6 and MIHA were purchased from American Type Culture Collection (Manassas, USA). Cells were cultured in DMEM supplemented with 10% FBS in a humidified atmosphere containing 10% CO_2_ and 90% air at 37°C. The medium was changed every three days, and cells were passaged using 0.05% trypsin/EDTA.

### MTT assay

The effect of HF on proliferation and viability of CRC cell lines and non-tumorigenic cell lines was determined by the 3-(4,5-dimethylthiazol-2-yl)-2,5-diphenyltetrazolium bromide (MTT) uptake method. Briefly, the cells (5,000 per well) were seeded in 96-well plates 24h prior to HF treatment. The effect of HF in CRC cell line proliferation was determined in different dosages and time points. Independent experiments were performed in triplicate.

### Colony formation assay

CRC cell lines were seeded in 6-well plates at a density of 1×10^5^ cells per well in 2 mL medium. Treatment with various concentrations of HF for 12 to 16 days until individual cells formed distinctly visible colonies. Thereafter, cells were stained with 50% methanol solution of 2% Methylene Blue. After washing, plates were air dried and digital images were taken. Independent experiments were performed in triplicate.

### Cell cycle analysis

The cell cycle phase distribution was determined by fluorescence-activated cell sorting (FACS) analysis of cellular DNA content. The cells were treated with different concentrations of HF for 12 h and then harvested by trypsinization and re-suspended in 200 μL PBS. The cells were fixed in 800 μL cold 100% ethanol, votexed, and then stored at −20°C overnight. The fixed cells were washed twice with PBS, re-suspended in 500 μL of PBS containing 5 μL RNase (10 μg mL^−1^) and incubated for 30 min in 37°C bath. The cells were stained with 50 μL propidium iodide (PI, 1 mg mL^−1^) for 5 to 10 min on ice in dark. The fluorescent signal was detected through the FL2 channel and the proportion of DNA in different phases was analyzed using ModFit LT version 3.1 (Verity Software House, Topsham).

### Annexin V/PI dye staining

SW480 and HCT116 cells were seeded in 6-well plates 24 h prior to HF treatment at a density of 5×10^5^ cells per well. After treated with different concentrations of HF for 12 h, apoptotic cells were assessed using the Annexin V-fluorescein isothiocyanate (FITC) apoptosis detection kit I following the manufacturer's instructions. Triplicate independent experiments were performed.

### Western blot analysis

After cells were treated with HF for 12 h, whole cell lysates were obtained by suspending the cells in lysis buffer. Followed by centrifugation at 13,500 rpm for 15 min at 4°C, total protein concentration was measured using Pierce(R) BCA Protein Assay Kit and 10 to 25 μg of protein was separated on 10% sodium dodecylsulphate-polyacrylamide gel (SDS-PAGE) and transferred onto polyvinylidene difluoride membranes. After blocking (5% skim milk powder in TBST, 20) for 1 h at room temperature, the membrane was then incubated with primary antibody overnight at 4°C. Antibodies against VDAC (D73D12), phosphor-mTOR (Ser2448), phosphor-p70S6 Kinase (Thr389), phosphor-4EBP1 (Thr37/46), phosphor-Akt (Ser473), Akt (pan) (C67E7), 4EBP1, p70S6 Kinase (49D7), mTOR (7C10), cleaved-PARP, cleaved-caspase 3, GLUT1 (D3J3A), fatty acid synthase (C20G5) and β-actin were purchased from Cell Signaling Technology, Inc. (Danvers, MA). The membrane was incubated with secondary antibody for 1 h at room temperature. HRP-goat anti-rabbit secondary antibody was purchased from Invitrogen (Carlsbad, USA). Goat anti-mouse IgG-HRP secondary antibody was purchased from San Cruz Biotechnology (Santa Cruz, USA). All antibodies were diluted in TBS-Tween 20 containing 5% dry milk. The immune-reactive proteins were detected by enhanced chemiluminescence (ECL) using X-ray film and ECL reagent.

### Quantitative analysis of mRNA levels

Total RNA was isolate from SW480 or HCT116 cells using TRIzol reagent (Invitrogen). cDNAs were prepared by reverse transcription and quantitative polymerase chain reaction (PCR) was performed using the Quantitect SYBR green PCR Master mix (Qiagen, Valencia, CA) with 1 μL cDNA in a final volume of 10 μL and the following primers at a final concentration of 1000 nM. Primers for Glut1 were 5′- TGGCATCAACGCTGTCTTCT-3′ (forward) and 5′- CTAGCGCGATGGTCATGAGT-3′ (reverse). Primers for G6PD were 5′- TGCATGAGCCAGATAGGCTG-3′ (forward) and 5′- GGTAGTGGTCGATGCGGTAG-3′ (reverse). Primers for HK-II were 5′- ACAAATTTCCGGGTCCTGCT-3′ (forward) and 5′- TGAGGAGGATGCTCTCGTCCA-3′ (reverse).

Amplification of Glut1, G6PD and HK-II cDNAs was performed using the LightCycler 2000 instrument (Roche, Indianapolis, IN). The cycling conditions comprised a denaturation step for 15 minutes at 95°C, followed by 40 cycles of denaturation (95°C for 15 seconds), annealing (59°C for 20 seconds), and extension (72°C for 15 seconds). After amplification, a melting curve analysis was performed with denaturation at 95°C for 5 seconds, then continuous fluorescence measurement from 70°C to 95°C at 0.1°C/second. Each sample was amplified in duplicate.

### Measurement of cellular ROS levels

Intracellular ROS levels were assayed using DCFH-DA as described previously [49]. Briefly, following exposure to different concentrations of HF for 12 h, CRC cells were counted to determine live cell concentration. Cells were then harvested by centrifugation at 250 g for 5 min and washed once with washing buffer. The cell pellet was then re-suspended in DMEM containing 20 μM DCFH-DA. Cell suspensions were then incubated for 30 min at 37°C under constant agitation. After washing twice with washing buffer, the cells were re-suspended in 1 mL of washing buffer (PBS containing 10 mM dextrose). Fluorescence was recorded using a 96-well plate reader operating at an excitation/emission wavelength of 485 nm/530 nm (EnVision 2104 Multilabel Reader, PerkinElmer). Mean fluorescence values of DCFH-DA-loaded cells were corrected by subtracting the autofluorescence background.

### Measurement of [NADP+]/[NADPH] Ratio

[NADP^+^]/[NADPH] ratios in CRC cell lines treated with HF were measured according to the protocol of [NADP^+^]/[NADPH] Quantification Kit (MAK038, Sigma). According to the NADPH standards, the concentration of NADP_total_ or NADPH can be expressed in pmole per 10^6^ cells. The ratio of [NADP^+^]/[NADPH] was calculated by ([NADP_total_] – [NADPH])/[NADPH].

### Xenograft studies

BALB/c nude mice, female, 6-week old, were obtained from the Laboratory Animal Services Centre, The Chinese University of Hong Kong. Mice were kept at room temperature 23 ± 2 °C with an alternating 12 h light-dark cycle, and were allowed access to food and water *ad libitum*. All of the experimental protocols were carried out with the approval of the Committee on Use of Human and Animal Subjects in Teaching and Research of Hong Kong Baptist University and according to the Regulations of the Department of Health, Hong Kong SAR, China. HCT116 cells (3 × 10^6^ cells/100 μL) were suspended in PBS and inoculated subcutaneously into the left flank of each mouse and tumor growth was monitored regularly. Once tumors were palpable, (∼100 mm^3^), mice were divided at random into two groups within 6 mice in each group: (i) Vehicle group, normally fed, receiving daily i.p. saline; (ii) HF group, normally fed, receiving daily i.p. HF (0.1 mg/kg/day) dissolved in 0.9% sodium chloride solution. The tumors were measured with calipers every 2 days, and the tumor volumes were calculated by the following formula: a^2^×b×0.4, where “a” is the smallest diameter and “b” is the diameter perpendicular to “a”. Other indicators of general health, such as body weight, feeding behavior, and motor activity of each animal were also monitored. After administration of HF or saline for two weeks, the mice were euthanized, and the tumor xenografts were immediately dissected, weighted, stored and fixed.

### Immunofluorescence assay and TdT-mediated dUTP nick end labelling (TUNEL) assay

Xenograft tumors were resected immediately and fixed in 10% neutral buffered paraformaldehyde at 4 °C for 24 h. Selected samples were embedded in paraffin, sectioned and stained with phosphor-Akt (Ser473), phosphor-mTOR (Ser2448), phosphor-p70S6 Kinase (Thr389), phosphor-4EBP1 (Thr37/46). All primary antibodies were used for dilution at 1:100. After overnight incubation at 4°C, the sections were incubated with flurochrome-conjugated secondary antibody for 1 h and stained with DAPI for 10 min. The sections were then mounted with DPX mountant (Sigma, 317616) for analysis. For the TUNEL assay, sections were deparafinnized and apoptotic cells were detected using the in situ BrdU-Red DNA fragmentation (TUNEL) assay kit (Abcam) and counterstained with DAPI.

### Metabolic flux analysis and lipidomics

[U-^13^C_6_]-glucose was used to feed cancer cells for metabolic flux analysis (MFA). Ultrahigh-performance liquid chromatography tandem mass spectrometry (UPLC-MS/MS) and gas chromatography mass spectrometry (GC/MS) were employed for MFA. Meanwhile, ultrahigh-performance liquid chromatography Orbitrap XL mass spectrometry was utilized for lipidomics analysis without labeling in cell culture. The details of metabolite extraction and chromatographic separation coupled with mass spectrometric conditions are described in the Supplementary Information.

## SUPPLEMENTARY MATERIAL FIGURES


