# Supplementary material for: Halofuginone inhibits colorectal cancer growth through suppression of Akt/mTORC1 signaling and glucose metabolism
Source: Oncotarget. 2015 Jun 8;6(27):24148–62. doi: 10.18632/oncotarget.4376 (PMC4695176; doi:10.18632/oncotarget.4376)
Supplement: Supplementary file 1 [file oncotarget-06-24148-s001.pdf]

Halofuginone inhibits colorectal cancer growth through suppression of Akt/mTORC1 signaling and glucose metabolism

Supplementary Material

Materials and methods

Cell culture for metabolic flux analysis

Human colon cancer cell line HCT116 was seeded into 10-cm dish at a density of 5×10^6 cells per dish in 5 mL medium, which maintained in high-glucose DMEM supplemented 10% FBS and 100 unites mL^{-1} penicillin-streptomycin. After cultured for 24 h, high-glucose DMEM was removed and cells were briefly rinsed with PBS twice. Then cells were cultured in DMEM (without glucose, L-glutamine, phenol red, sodium pyruvate and sodium bicarbonate) with 4 mM label-free glutamine,10% dialyzed FBS and 100 unites mL^{-1} penicillin-streptomycin and 25 mM [U-$^{13}C_6$]-glucose for 12 h. Meanwhile, 20 nM HF was added in the medium compared to control group without HF treatment.

Metabolite extraction

After 12 h-treatment of HF in cancer cells, culture medium was removed into a new tube. Cells were rapidly rinsed with PBS twice. The residual PBS was removed by vacuum. Cells were then quenched using 1.5 mL cold HPLC-grade methanol. Next, cells were quickly detached from the culture dish using a cell lifter. The methanol solution containing the quenched cells was pipetted into a 2-mL centrifuge tube. Samples were then vortexed-mixed for 30 sec and submerged for 1 min in liquid nitrogen. Samples were then thawed in ice. This process was repeated three times and samples were centrifuged at 10,000g for 10 min at 4 °C. The supernatant was removed into a new tube and cold 0.5 mL 80% methanol was added to the

residue for vortexed-mixed for 30 sec, and samples were centrifuged at 10,000 g for 10 min at 4 °C. Then the two extraction solutions were pooled together in a tube for evaporation till dryness under airflow and stored at –80 °C till analysis.

UPLC-MS/MS analysis

Two hundred microliter of cold solvent mixture (ACN: MeOH: H_2O, 40:40:20, v/v/v) was added to the dried residue. The mixture was vigorously vortexed for 30 s and stored at –20 °C for 1 h, then centrifuged at 14,000 g for 15 min at 4 °C. The 150 μL supernatant of each sample was transferred to a new glass tube for UPLC-MS/MS analysis.

Analysis was performed on a TSQ Quantum Ultra triple quadrupole mass spectrometer (Thermo Fisher Scientific) via an electrospray interface (ESI), operating in negative ionization mode and configuring in selective reaction monitoring (SRM) mode. The metabolite separation was performed using an ACQUITY UPLC® BEH Amide (1.7 μm, 100×2.1 mm) column (Waters, Ireland). The mobile phases were consisted of acetonitrile (A) and 20 mM ammonium formate and 20 mM ammonium hydroxide in solvent mixture (water:acetonitrile, 95/5, v/v) (B). The gradient elution program initiated from 80% A; decreased to 65% A in 4 min; to 60% A in 6 min; to 55% A in 8 min; to 5% A in 10 min; and held for 13 min with a flow rate of 0.3 mL min^{-1}.

Mass spectrometric conditions were optimized for each metabolite by using reference standard. Spray voltage, vaporizer temperature, sheath gas, auxiliary gas and capillary temperature were set 2800 V, 350 °C, 35 arb, 8 arb and 300 °C, respectively. The LC-MS/MS data were acquired and processed with LCquan™ software version 2.5.6 (Thermo Fisher Scientific).

GC/MS analysis

An internal standard (10 μL L-4-chloro-phenylalanine in water, 5 μg mL^{-1}) was added to the residue, mixed and freeze-dried. Subsequently, 80 μL of methoxylamine solution (15 mg mL^{-1} in pyridine) was added to each vial. The resultant mixture was vortex-mixed for 1 min and reacted at 37 °C for 24 h in order to inhibit the cyclization of reducing sugars and the decarboxylation of R-keto acids. Eighty μL BSTFA (with 1% TMCS) were added into the mixture and derivatized at 70 °C for 60 min, and vortexed-mix for 30 sec and samples were centrifuged at 10,000 g for 10 min at room temperature. The supernatant was removed to a new glass tube prior to analysis.

The derivatives were separated on a GC column DB-5MS fused-silica capillary column (30m × 250μm i.d., 0.25um film thickness, Agilent J&W Scientific, Folsom, CA). Helium as a carrier gas was used at a constant flow rate of 1 mL min^{-1}. One μL of derivative was injected, and the solvent delay time was set to 5.5 min. The initial oven temperature was set at 60 °C for 2 min, ramped to 280 °C at a rate of 10 °C min^{-1}, and finally held at 280 °C for 6 min. The temperatures of injector, transfer line, and electron impact ion source were set at 250 °C, 280 °C and 230 °C, respectively. The initial inlet gas pressure was 8.2317 psi and electron energy was 70 eV. Mass data was collected in a full scan mode from 6.5 to 28 min and the *m/z* range was set at 50 to 600.

Cell culture for lipidomics analysis

Human colon cancer cell line HCT116 was seeded into 10-cm dish at a density of 5×10^6 cells per dish in 5 mL medium, which maintained in high-glucose DMEM supplemented 10% FBS and 100 unites mL^{-1} penicillin-streptomycin. After cultured for 24 h, cells were treated with 20 nM HF for 12 h, and then removed the culture medium by vacuum. Cells were rapidly rinsed

twice with PBS, then adding 1 mL PBS to the dish and quickly detached from the dish using a cell lifter. Removed the liquid containing cells into a 2-mL tube and centrifuged at 10,000 g for 10 min. The supernatant were discarded and adding 400 μL cold 80% methanol (20% water) to mix cells. The mixing cells were cracked by ultrasonic extraction 2 min, and then added 1 mL Methyl tert-butyl ether (MTBE) into the tube. After shaking at room temperature for 1 h, added 250 μL water and placed 10 min, then centrifuged at 10,000 g for 10 min. The organic phase is in the upper supernatant and the water phase is in the under layer. The organic phase was transferred to a new tube and dried under gentle nitrogen stream. The water phase was also transferred to a new tube and stored at –80 °C till analysis.

UPLC/LTQ-Orbitrap MS for lipidomic analysis

Thermo Fisher Accela 1250 UHPLC coupled online via ESI with an LTQ Orbitrap XL (Thermo Fisher Scientific) hybrid mass spectrometer was employed for lipidomic analysis with modification from previous literatures. External mass calibration of the Orbitrap prior to sample analysis was performed by flow injection of the calibration polytyrosine-1, 3, 6 solution according to the manufacturer’s instruction. Sample aliquots were reconstituted in 200 μL solvent mixture (ACN: isopropanol: water, 65:30:5, v/v/v). A QC sample was prepared by pooling 50 μL from all of the control group and treatment group of each cell line. To cover different lipid species, chromatographic separation was performed on a reversed phase UPLC ACQUITY BEH C18 column (2.1 mm×100 mm×1.7 μm) (Waters, Milford, USA) by gradient elution. Mobile phase A was 60% ACN in water containing 10 mM ammonium acetate and 0.1% acetic acid, and B was isopropanol:ACN (9:1), containing 0.1% acetic acid. The flow rate was 0.2 mL min^{-1}, with the gradient elution program as follows: 25% B held for 1 min, then linearly

increased to 70% B from 1 to 4 min, then to 97% B from 4 to 15 min and held for 8 min followed by equilibration with 25% B for 6.5 min. Mass spectrometric detection was performed in positive ion mode with ESI. High resolution data (resolution 30,000) was acquired by full scan from *m/z* 450 to 1500 with source voltage of 3500 V, capillary temperature of 300 °C, sheath gas flow of 40 arb, auxiliary gas flow of 5 arb, ion spray temperature 350 °C and tube lens of 110 V. Prior to sample analysis external mass calibration was applied to ensure mass accuracy of the mass spectrometer.

Immunohistochemical staining

Paraffin-embedded tumors were sectioned (4μM) and mounted on glass slides, deparaffinizedin xylene and rehydrated in decreasing concentrations of ethanol (from 100% to 70%). For antigen retrieval, slides were heated in sodium citrate buffer (10 mM citric acid) for 15 min. Endogenous peroxidase was blocked by incubation with 3% H_2O_2 (Dako, Ely, Cambridgeshire, UK) at room temperature for 30 min before incubation with phosphor-Akt (Ser473), phosphor-mTOR (Ser2448), phosphor-p70S6 Kinase (Thr389), phosphor-4EBP1 (Thr37/46) antibodies overnight. After washing with PBS, the sections were incubated with horseradish peroxidase-conjugated secondary antibodies for 1 hour and diaminobenzide (Dako) was used as the chromogen.

Figure S1.

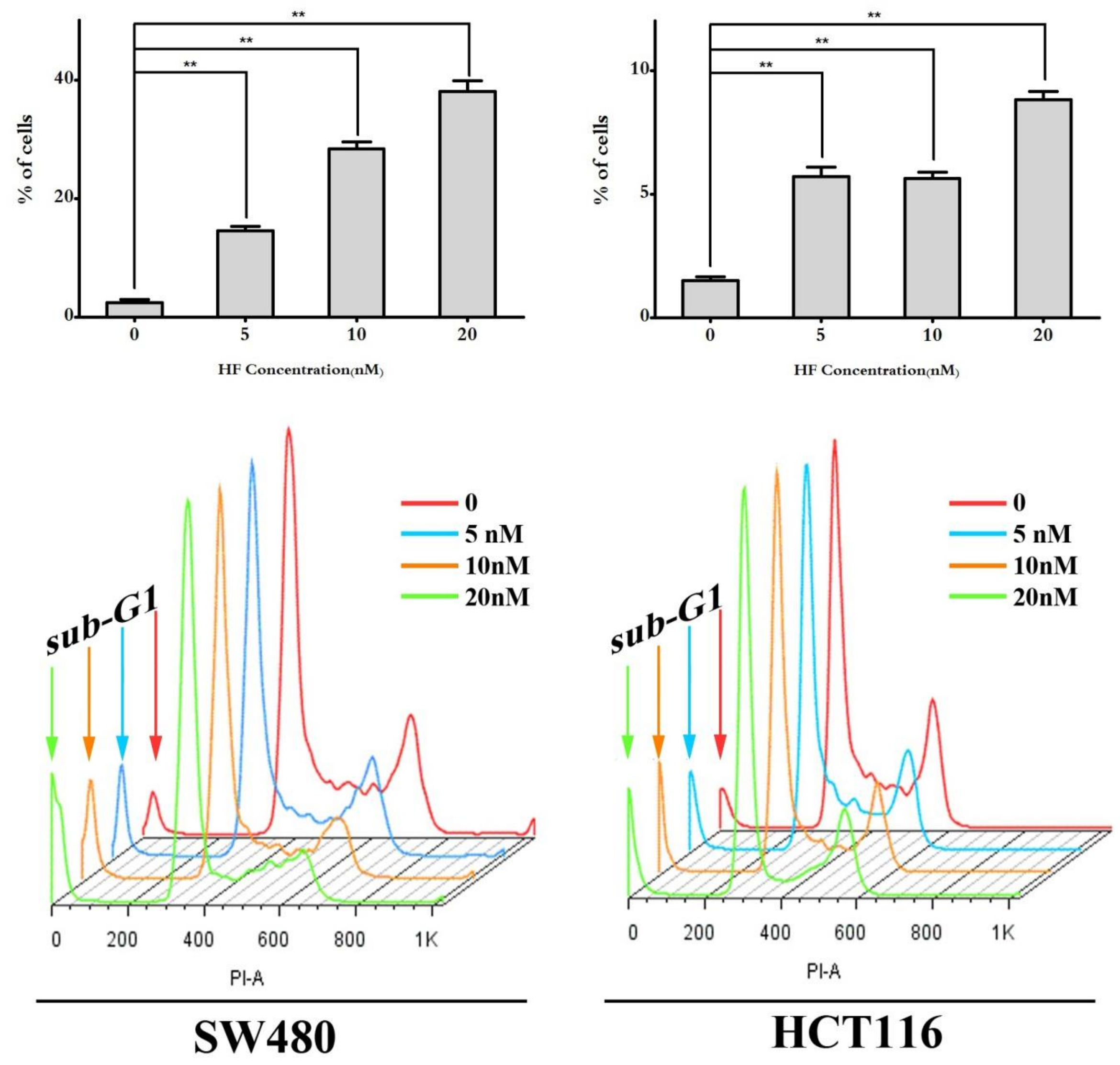

Figure S1: Sub-G1 percentage indicates that the cytotoxic effect of HF-induced apoptosis in SW480 and HCT116 cell lines. The percentage of sub-G1 in the different phases of cell cycle regulation (upper panel), and the sub-G1 peaks showed in the cell cycle distribution of both cell lines (lower panel). * $P < 0.05$, ** $P < 0.01$, compared with control group.

Figure S2.

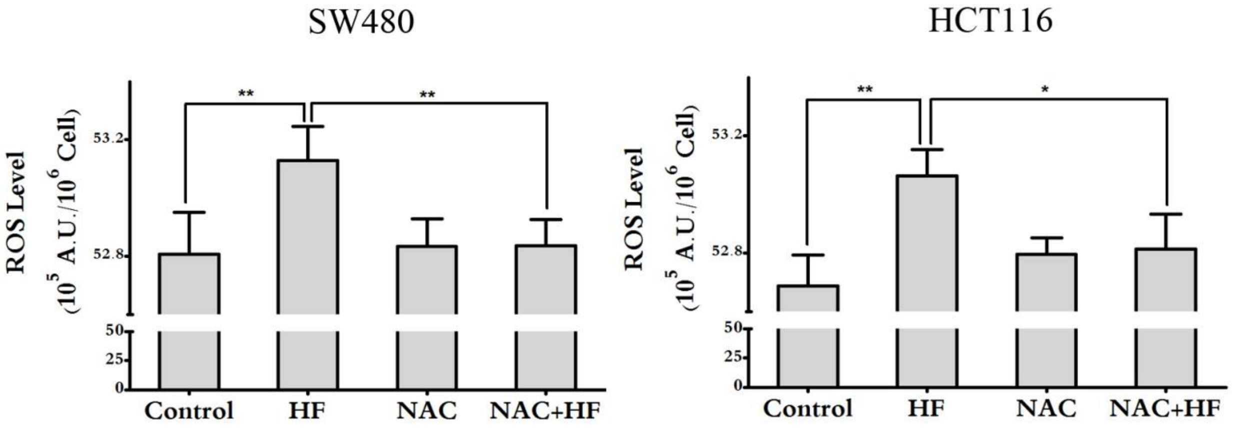

Figure S2: N-Acetyl-L-cysteine reverses the ROS levels in SW480 and HCT116 cell lines upon halofuginone treatment. * $P < 0.05$, ** $P < 0.01$ in the comparison between control and HF treatment or HF treatment and co-treatment of HF and NAC.

Figure S3.

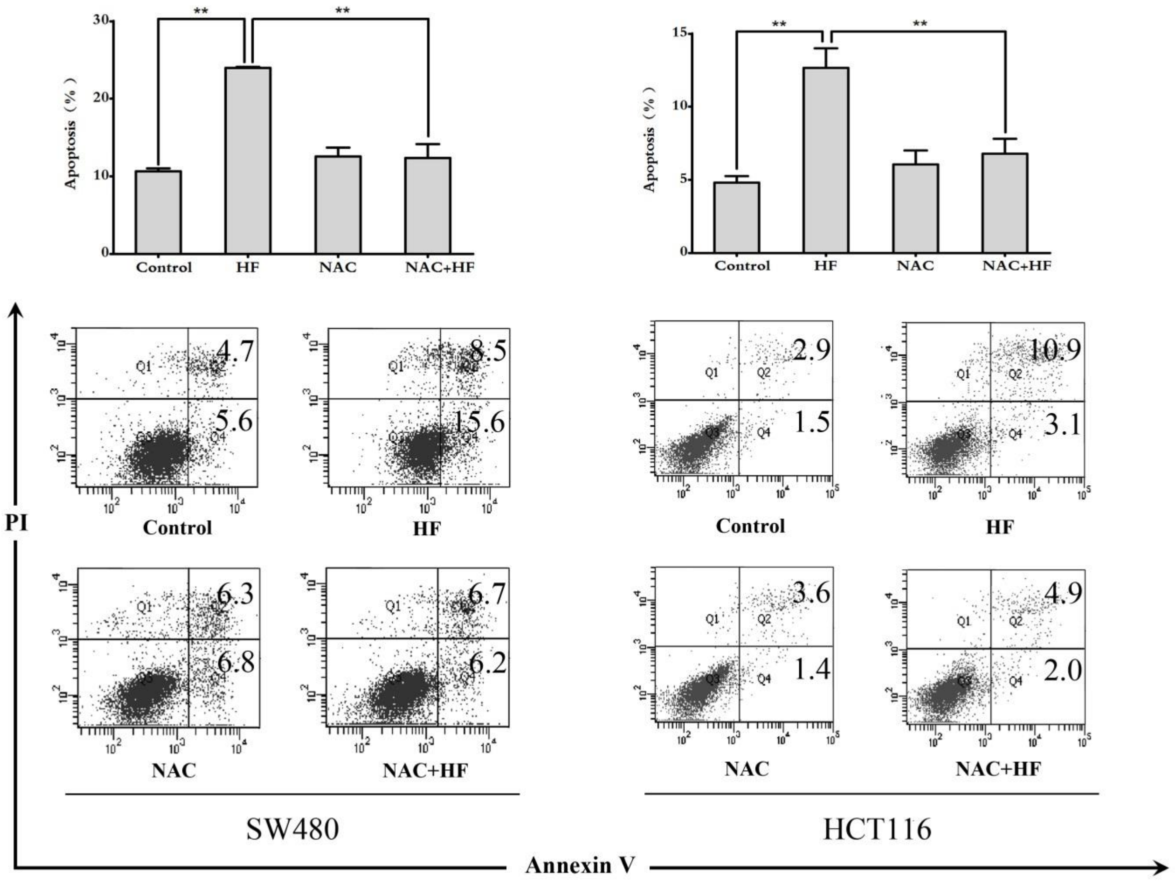

Figure S3: N-Acetyl-L-cysteine reverses the effect of HF-induced apoptosis by a flow cytometry annexin V-PI assay. ** $P < 0.01$ in the comparison between control and HF treatment or HF treatment and co-treatment of HF and NAC.

Figure S4.

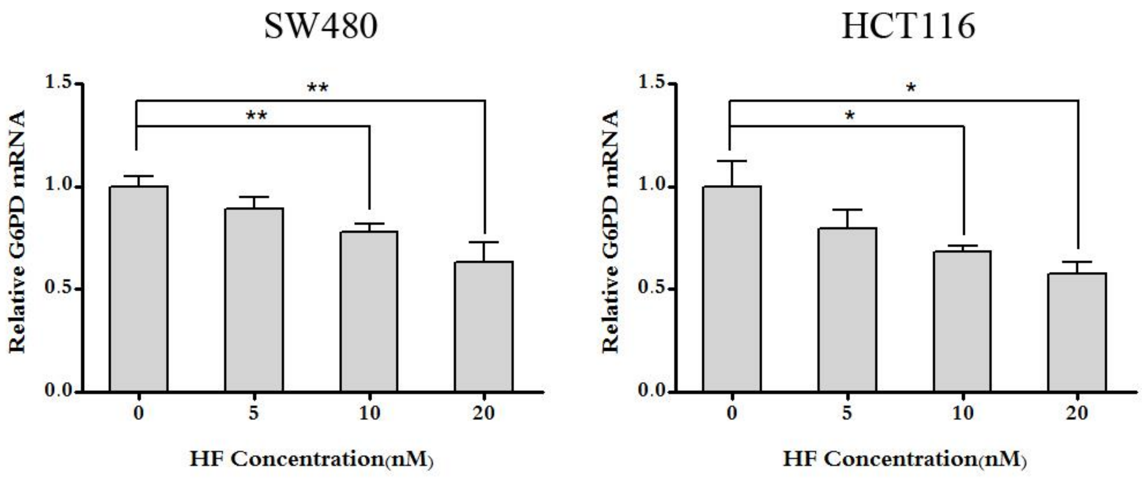

Figure S4: Quantitative RT-PCR analysis of G6PD mRNA in SW480 and HCT116 cell lines treated with 0, 5, 10, 20 nM of halofuginone. * $P < 0.05$, ** $P < 0.01$, compared with control group (0 nM of HF).

Figure S5.

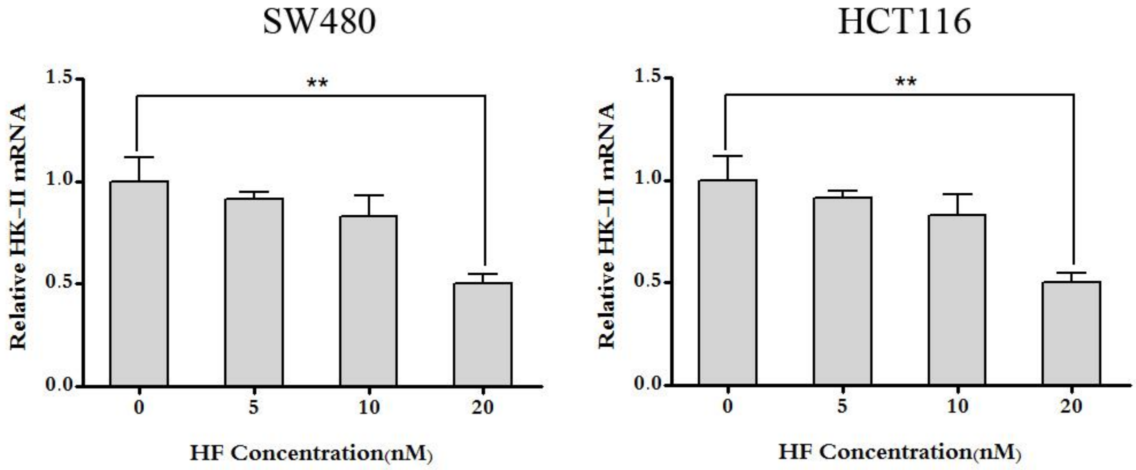

Figure S5: Quantitative RT-PCR analysis of HK-II mRNA in SW480 and HCT116 cell lines treated with 0, 5, 10, 20 nM of halofuginone. ** $P < 0.01$, compared with control group (0 nM of HF).

Figure S6.

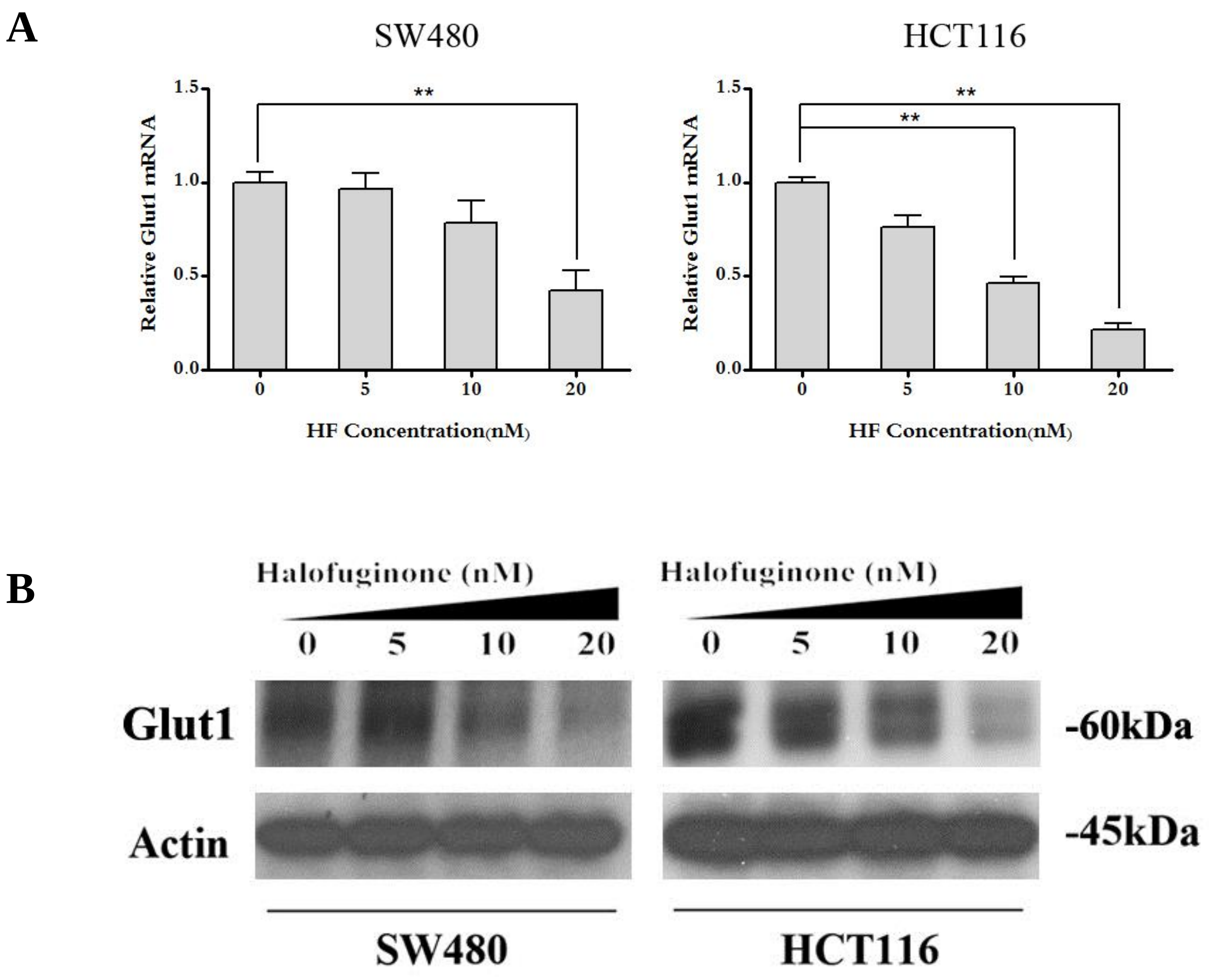

Figure S6: Halofuginone inhibits glucose transporter GLUT1 in CRC cells. (a) Quantitative RT-PCR analysis of GLUT1 mRNA in SW480 and HCT116 cell lines treated with 0, 5, 10, 20 nM of halofuginone. (b) Protein expressions of GLUT1 in SW480 and HCT116 cell lines treated with 0, 5, 10, 20 nM of halofuginone. ** $P < 0.01$, compared with control group (0 nM of HF).

Figure S7.

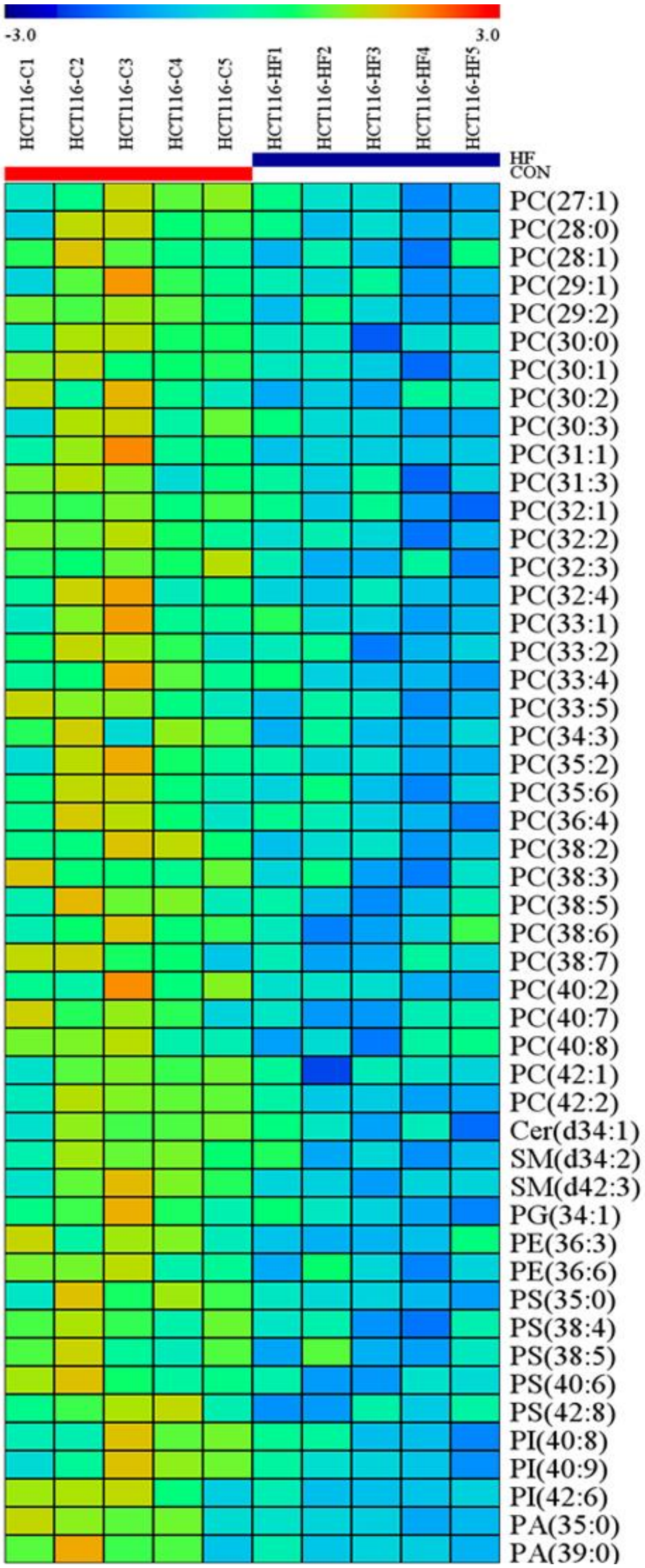
-3.0
3.0
HCT116-C1
HCT116-C2
HCT116-C3
HCT116-C4
HCT116-C5
HCT116-HF1
HCT116-HF2
HCT116-HF3
HCT116-HF4
HCT116-HF5
HF
CON
PC(27:1)
PC(28:0)
PC(28:1)
PC(29:1)
PC(29:2)
PC(30:0)
PC(30:1)
PC(30:2)
PC(30:3)
PC(31:1)
PC(31:3)
PC(32:1)
PC(32:2)
PC(32:3)
PC(32:4)
PC(33:1)
PC(33:2)
PC(33:4)
PC(33:5)
PC(34:3)
PC(35:2)
PC(35:6)
PC(36:4)
PC(38:2)
PC(38:3)
PC(38:5)
PC(38:6)
PC(38:7)
PC(40:2)
PC(40:7)
PC(40:8)
PC(42:1)
PC(42:2)
Cer(d34:1)
SM(d34:2)
SM(d42:3)
PG(34:1)
PE(36:3)
PE(36:6)
PS(35:0)
PS(38:4)
PS(38:5)
PS(40:6)
PS(42:8)
PI(40:8)
PI(40:9)
PI(42:6)
PA(35:0)
PA(39:0)

Figure S7: Heat map displays fold changes of lipid species in HCT116 cells between HF-treated group and control group. (HF: halofuginone; CON: control group). (PC: phosphatidylcholine, Cer: ceramide, SM: sphingomyelin, PG: phosphatidylglycerol, PE: phosphatidylethanolamine, PS: phosphatidylserine, PI: phosphatidylinositol, PA: phosphatidic acid)

Figure S8.

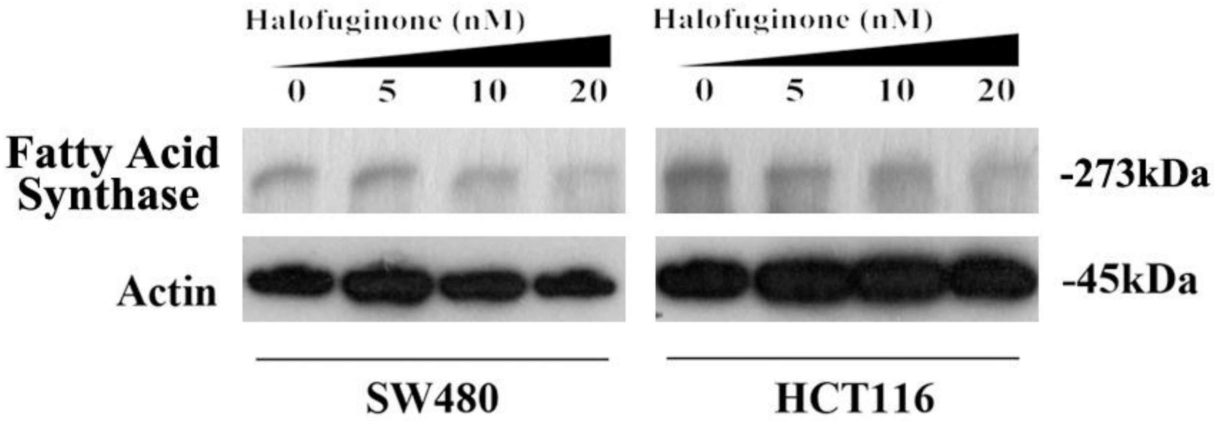

Figure S8: Protein expressions of fatty acid synthase in SW480 and HCT116 cell lines treated with 0, 5, 10, 20 nM of halofuginone.

Figure S9.

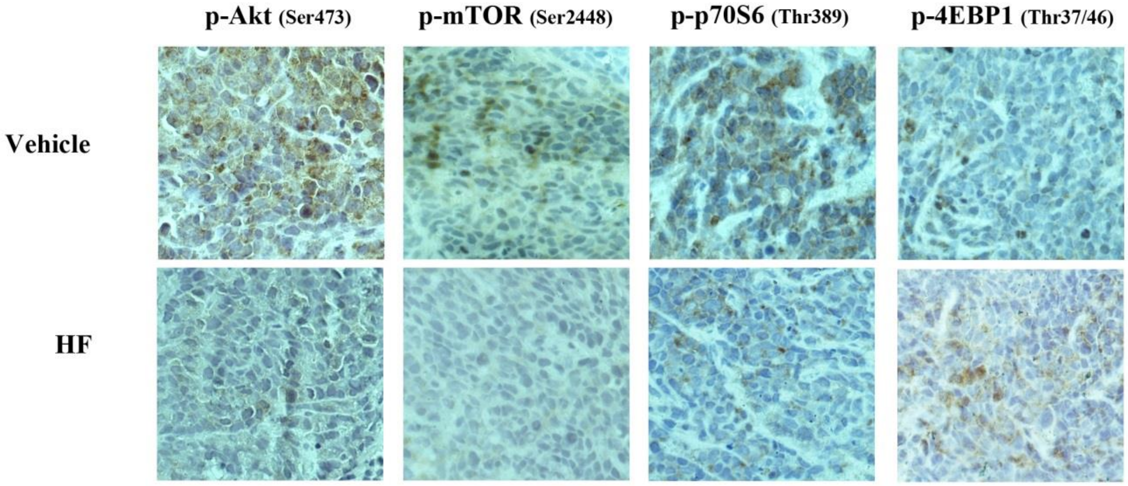

Figure S9: Immunohistochemical staining of tumor tissue samples in nude mice by incubating with primary antibodies of p-Akt, p-mTORC1, p-p70S6K and p-4EBP1.
